# The Effects and Mechanisms of Phytochemicals on Pain Management and Analgesic

**DOI:** 10.3390/nu17040633

**Published:** 2025-02-11

**Authors:** Milan Patel, Sayed Wahezi, Philippe Mavrocordatos, Alaa Abd-Elsayed

**Affiliations:** 1 Department of Anesthesiology, University of Wisconsin School of Medicine and Public Health, Madison, WI 53792, USA; mapatel5@wisc.edu; 2 Department of Pain Managment, Montefiore Medical Center, Bronx, NY 10461, USA; 3 Swiss Pain Institue, 1003 Lausanne, Switzerland

**Keywords:** phytochemical(s), pain management, hyperalgesia, mechanism(s), inflammation, inflammation, pro-inflammatory cytokines, nociception

## Abstract

Phytochemicals can be an essential treatment for chronic pain. This narrative review will summarize and critically analyze the evidence surrounding these substances in pain management. We will introduce phytochemicals, discuss their associated mechanisms, and comment on their viability for potential treatment. There have been decades of research on phytochemical therapies for pain management, but the authors limited the scope of the investigation to the last 25 years. This literature review will serve as a foundation for the pain practitioner to understand where these treatments fit in the paradigm for chronic pain treatment. Assess the integration of phytochemicals within pain management fully.

## 1. Introduction

Biologically active plant metabolites used for clinical or wellness purposes are termed phytochemicals. Much of the literature has been on animal models in oncologic translational medicine settings and [1,2]. However, further testing on more extensive and more animal populations must be carried out to support this initial finding. According to Shin et al., the additional uses of phytochemicals have been an interesting topic of question, and more research continues to come out regarding the usage and application of phytochemicals not only for cancer but potentially for pain management. Phytochemicals have been an increasingly popular topic of conversation as their application in pain management is also further studied.

Phytochemicals can serve as a potential alternative or adjuvant to anti-inflammatory medication, making them a possible therapy for chronic pain patients. However, the extent to which these medications can be prescribed is limited due to the side effects that accompany them. Thus, phytochemicals are subject to further research and a point of interest regarding their anti-inflammatory properties. Animal models and human populations are being tested to understand the efficacy and tolerability of phytochemicals compared to pharmaceuticals [3].

This review will begin with a general overview of the broader category in which the consequent phytochemicals mentioned will be a part. The broader categories will be broken down into polyphenols, terpenoids, and alkaloids. Each of these categories will have phytochemicals mentioned after them, which this review will go into further detail on. After the last phytochemical, the review will transition towards diving into pathways and inflammatory mediators that play crucial roles in inflammation. The review will begin with the nociceptive pathway followed by the inflammatory mediators. The inflammatory mediators discussed will include pro-inflammatory cytokines TNF-alpha and Interleukins-1Beta/6, inflammatory enzymes COX and NOS, and lastly, inflammatory pathways NF-kB and MAPK. This review will end with future directions and suggestions for research and physicians involved with phytochemicals.

### Nociceptive Signaling and Receptors

Nociceptive signaling is one of the main mechanisms of pain management. Nociceptors are afferent sensory neurons in peripheral and visceral areas as well. These are pseudounipolar neurons. The two neuron branches are indistinguishable biochemically, and most proteins produced here are distributed to the central and peripheral terminals [4].

Inflammatory mediators activate the nociceptors. The inflammatory mediators are released during tissue injuries, depolarizing the nociceptive receptors. This is summarized as hyperalgesia. When tissue damage or injury occurs, inflammatory mediators will be released from resident cells, infiltrated cells, and activated nociceptors [4].

Nociceptive signaling is one of the most common chronic pain mechanisms seen by physicians and causes a multitude of injuries to patients. Some pro-inflammatory agents act through cell surface receptors that are on nociceptors. This is where many pathways that cause activation in the form of hypersensitivity of the nociceptors begin, and thus, the inflammatory mediators are targets for pain reliever development [4].

Looking closer at TRPV1, which is activated by noxious heat, reduced pH, and capsaicin, it can be compared to TRPV2. TRPV2 and TRPM8 respond more to cold methanol, while mustard and garlic activate TRPA1 and KNCK channels [5]. Several ion channels are heavily involved in thermal and mechanical pain sensitization. The modulation of TRPV1 is considered an essential factor when examining chronic pain and inflammation. Studies have shown that TRPV1 functions to integrate multiple signals and that thermal stimuli sensitivity can be significantly altered [4,6].

The TRPV1 channel is the most important channel out of the TRPV family for this review. Various stimuli can activate the TRPV1 receptor; however, capsaicin is the main phytochemical we will discuss in this review [7]. According to González-Ramíerez et al., TRPV1 can be expressed in both the CNS and PNS. Specifically, the small and medium nociceptor neurons will have the most significant effect in the PNS, with some coming from the DRG [7]. In the CNS region, TRPV1 is expressed in many areas; however, the laminae I and II of the dorsal horn of the spinal cord are the most important for our purposes [7].

TRPV1 is most effective in normal conditions around 42 degrees Celsius. When assessing TRPV1 concerning inflammation, the thermal threshold decreasing to around 35–37 degrees Celsius is important to consider after the medium is acidified.

Besides post-translation modifications like phosphorylation, MAPK also increases TRPV1 expression [7]. I will go further into MAPK pathways later in this review article.

Regarding inflammatory pain, IL-1beta, IL-6, and TNF-alpha mediators are generated when a lesion is present. TRPV1 can integrate multiple pro-inflammatory cytokines and mediators; thus, testing phytochemicals such as capsaicin in rodent models is increasingly more important [7].

There are very few direct mediators of these channels that lead to a decreased activation threshold for conditions such as hyperalgesia. TRPV1 is a point of interest because of its potential benefits in pain relief regarding infections, osteoarthritis, rheumatoid arthritis, and inflammatory bowel disease [4]. TRPV1 will be a critical point of further research as it plays a significant role in inflammation.

## 2. Classifications of Phytochemicals

### 2.1. Polyphenols

Polyphenols are the most abundant of antioxidants in humans. However, this review will focus more closely on flavonoids and nonflavonoids. Flavonoids are a sub-category of the polyphenol family and can come in more than 6000 structures [8]. Generally, the structure of flavonoids will contain two phenyl rings, which are connected by a heterocyclic pyrene ring with an oxygen atom attached [8]. However, there is a counterpart called the nonflavonoids. The nonflavonoid group will be classified based on the carbons present and the attached subgroups [9]. The purpose and usage of polyphenols such as curcumin, resveratrol, and quercetin will be discussed later in this review, as well as their potential health benefits [10].

#### 2.1.1. Curcumin

Curcumin is a bright yellow compound driven by the Curcuma longa Linn plant. However, curcumin has been further researched and is widely known to be a great pain reliever. Initial studies have suggested that curcumin has potent anti-inflammatory and antioxidant properties. The current literature suggests that it may be applicable in cases of inflammation management for osteoarthritis and rheumatoid arthritis [11]. According to Bisht et al., curcumin inhibits the COX-2, NF-kB, inducible NOS, and NO enzymes in specific macrophages and natural killer cells. Also, it describes curcumin as having inhibitory properties on specific pro-inflammatory cytokines. These cytokines consist of but are not limited to, TNF-alpha, IL-1Beta, and IL-6 [12].

Furthermore, there have been results in rodent studies showing the antihyperalgesic effects of curcumin in rodents by affecting the TNF-alpha, IL-1Beta, and IL-6 pro-inflammatory cytokines. Singh et al. conducted a rodent study that found that curcumin decreased the effects of TNF-alpha, IL-1Beta, and IL-6 [4]. Singh et al. reported that curcumin is possibly linked to the inhibition of nitric oxide species, TNF-alpha release, COX-2, and 5-LOX inhibition, as well as capsaicin’s TRPV1 receptor due to the similarities in structure [4].

The main findings in both studies present opportunities for further testing to solidify the multipurpose potential of curcumin. Increasing the usage and reliability of phytochemicals for chronic pain management use could lead to more excellent patient outcomes, with fewer side effects being a contributing factor.

#### 2.1.2. Resveratrol

Resveratrol is a white powder predominantly found in grapes and red wine, which has anti-inflammatory effects with potential chronic pain management applications. Resveratrol can inhibit NF-kB and TNF-alpha [12]. Resveratrol, in systematic dosages, can be antinociceptive. In addition to the antioxidant properties of resveratrol, it has also been shown to be another potential solution [4]. Curcumin and resveratrol have applications now, as discussed, regarding their anti-inflammatory and antioxidant capabilities. However, more studies need to be published to confirm these results. More studies within a more recent timeframe would help establish concrete results and provide a path for these phytochemicals to be tested in potentially large-scale human populations.

#### 2.1.3. Quercetin

Quercetin, a flavonoid, has emerged as a promising phytochemical in pain management with its anti-nociceptive effects [13]. According to Liu et al., quercetin has neuroprotective effects in rat models [14]. Liu et al. describe a rat model with a spared nerve injury (SNI)-induced neuropathic pain, which showed that the oral administration of quercetin reduced pain in L5 dorsal root ganglions through satellite glial cell inhibition [13,14]. This finding is crucial as it builds onto curcumin and resveratrol, phytochemicals that can provide anti-inflammatory and neuroprotective effects through pathway and glial cell inhibition.

Liu et al. also noted that quercetin was able to provide analgesic effects through the inhibition of nociceptive effects of IL-1Beta and TNF-alpha in rodent models [13]. As I will describe further in this review, IL-1Beta and TNF-alpha play significant roles in the mechanisms of chronic pain manifestation.

In rodent model studies analyzed by Takeda et al., quercetin was seen as a viable pain relief treatment option in neuropathic pain models. Using the COX-2 pathway, quercetin resulted in a decrease in the mechanical stimulus threshold in rat models. Noteworthily, there was an increase in the frequency of spontaneous and evoked discharges. However, all central sensitization signs returned to normal after a short period after the quercetin was administered [15]. Quercetin has shown great potential as a pain management treatment in rat models and should be further tested for its applications in humans. Quercetin, resveratrol, and curcumin have all shown promising effects with their anti-inflammatory and antioxidant properties. However, further studies in rodent populations should be carried out to support the current findings.

#### 2.1.4. Naringenin

This phytochemical is both bitter and colorless in nature. The antihyperalgesic potential of naringenin has been discovered more recently than most of the other phytochemicals mentioned. Naringenin, also a polyphenol, is associated with flavonoids and, similarly to the other phytochemicals previously mentioned, has also emerged as a player in chronic pain management. This phytochemical also displays antioxidant and neuroprotective effects as described by Motallebi et al. [16]. According to Motallebi et al., naringenin exhibits its neuroprotective capabilities through the modulation of ROS and raises superoxide dismutase levels in chronic diseases. Furthermore, Motallebi et al. support their review by stating that naringenin showcases anti-inflammatory effects by decreasing cytokine production in macrophages and dendritic cells. Motallebi et al. recorded their findings through in vivo and in vitro studies. Narigenin, similarly to the other phytochemicals mentioned, displayed inhibitory effects of inflammatory mediators such as IL-1Beta [16].

The anti-hyperalgesic effects of naringenin have been reported in diabetic neuropathy studies. The mechanism is believed to be modulation of the antioxidant enzyme superoxide dismutase (SOD). Naringenin has also been shown to affect ROS-dependent downstream signaling related to hyperalgesia [4]. During this literature review, I found less updated literature on the effects of naringenin, thus warranting more testing on this phytochemical effect on additional rodent populations. While the initial results are promising, similar to the other phytochemicals, this one is more so; however, more research is needed to confirm and support the initial findings before clinical applications in humans can be justified.

#### 2.1.5. Epigallocatechin Gallate (EGCG)

Epigallocatechin gallate is another phytochemical that has excellent potential pain management applications. It is most commonly found in dried leaves of green tea and the most abundant catechin in tea. EGCG is a polyphenol and is known to exhibit neuroprotective effects in a multitude of pathological states. ECGC is also most commonly known for its antioxidant properties, and studies have widely referred to it as the most involved property in pain management [4].

In a study by Yuan et al., the phytochemical epigallocatechin gallate was tested on an obese rodent population to observe whether the phytochemical provided health benefits to the rodents [17]. The importance of finding solutions to chronic pain and inflammation issues through solutions with fewer side effects is a point of urgency. After close monitoring of the rodents upon giving a set dosage of epigallocatechin gallate, Yuan et al. were able to see results regarding their overall question of extending the life span of these rodents. Yuan et al. reported that their rodents’ primary sources of oxidative stress and inflammation came from IL-6, TNF-alpha, and ROS, which showed a decrease upon epigallocatechin gallate being introduced to the population. Thus, they claimed that EGCG was the direct cause of this decrease in inflammation and oxidative stress in the population, contributing to the idea that this phytochemical could provide better relief than previous methods [17].

Researchers have also questioned the ability of EGCG’s antioxidant properties to aid neuropathic pain relief. Furthermore, there is potential for ECGC to be used to decrease diabetes-induced neuropathic pain, as seen by lower spinal ROS mentioned by Singh et al. in their review. Intrathecal injection of EGCG has been shown to improve pain relief in CCI-induced neuropathic pain. Singh et al. note that this is in conjunction with decreased TLR4, NfxB, HMGB1, TNF-alpha, and IL-1Beta. Consequently, there is increased IL-10 in the spinal cord [4]. I would like to see more studies confirming the decreased levels of the inflammatory mediators mentioned by Singh et al. EGCG would have great application if larger-scale studies and additional studies confirmed the findings. EGCG would benefit more as it is the most abundant catechin [4].

### 2.2. Terpenoids

Terpenoids are another group of phytochemicals linked to human health benefits. As another group of phytochemicals with anti-inflammatory and antioxidant properties, testing their ability within chronic pain applications has been a growing idea. Thus, Masyita et al. stated in their review that the application of terpenes and terpenoids has successfully been shown to affect neuroinflammation [18]. Masyita et al. also state that terpenoids have demonstrated anti-inflammatory capabilities in rodent models’ previously mentioned pathways and mechanisms, such as IL-1Beta, IL-6, and TNF-alpha [18]. Terpenoids have shown initial success. Further testing will be required to confirm these results. In the next section, I will describe how Eugenol can relieve pain and its ability to be an analgesic as a terpenoid.

Many living organisms can create terpenes for essential physiological functions. The variety of terpene structures that can be produced has allowed for great diversity within this classification. Terpenoids have well-described characteristics and have a functional purpose in almost all parts of plant development. Terpenes assist with growth, development, reproduction, and defense [8,9].

#### Eugenol

This phytochemical can be colorless or a pale yellow, oily liquid that can be extracted from essential oils. Eugenol can be found in nutmeg, cinnamon, basil, and bay leaves. Eugenol, a type of terpenoid, serves as another phytochemical with potential chronic pain management application. Like the previously mentioned phytochemicals, eugenol also possesses anti-inflammatory and antioxidant properties utilizing the interleukin and tumor necrosis factor pro-inflammatory cytokines. Nisar et al. describe eugenol’s antioxidant, anti-inflammatory, and neuroprotective effects in their review, showcasing studies of eugenol in action [19]. Nisar et al. state that eugenol in a Swiss albino mice population reduced inflammation markers of COX-2, iNOS, TNF-alpha, and antioxidant enzymes [19].

According to Singh et al., eugenol has also shown antibacterial, antifungal, and anti-inflammatory actions. This is accomplished by inhibiting specific inflammatory mediators such as COX-2, IL-1Beta, and TNF-alpha. Singh et al. also state that eugenol can also be used to inhibit voltage-gated sodium channels and DRG neurons in rats [4]. These findings are significant as eugenol could have nociceptive pain pathway implications with the ability to affect ion channels. Eugenol should be further tested as the initial results are promising, and its effect on ion channels differs from the literature on other phytochemicals I have found.

### 2.3. Alkaloids

Alkaloids, as the name hints, display properties similar to inorganic alkalis. They are approximated to be in around 20% of plants, and their primary function stems from plant defense [8]. Alkaloids comprise nitrogen atoms (basic) and other weakly acidic compounds. Also, alkaloids can be found in many areas, including animals, bacteria, fungi, and plants. Similarly to the different categories of phytochemicals, alkaloids also possess the ability to provide pain relief [20]. In the next section, I will provide information on how capsaicin acts as an analgesic and the potential of capsaicin as a chronic pain management treatment option.

#### Capsaicin

Capsaicin is the main ingredient in hot peppers, including hot chili peppers. Similarly to curcumin, capsaicin has also been used historically for pain management. Capsaicin can bond to the transient receptor potential vanilloid 1 (TRPV1), the main mechanism of action capsaicin utilizes [16,21]. Prolonged activation of TRPV1 by capsaicin in conjunction with enzyme or osmotic changes has resulted in loss of receptor functionality [4]. According to Reyes-Escogido et al., capsaicin has gained more traction as a human analgesic. In rat models and human clinical trials, capsaicin has been identified as a validated track record for a wide range of painful peripheral neuropathic conditions.

Table 1 differentiates the type of phytochemical present as well as the main components. The grouping of phytochemicals in the table can allow for contrast and comparisons between each other. The components associated with each phytochemical are those of primary significance and include various inflammatory mediators (proinflammatory cytokines, inflammatory enzymes, and inflammatory pathways).

## 3. Inflammatory Mediators

### 3.1. Pro-Inflammatory Cytokines

#### 3.1.1. Tumor Necrosis Factor-Alpha (TNF-α)

Tumor necrosis factor-alpha (TNF-alpha) is a potent pro-inflammatory cytokine. It has roots tracing back to 1975 when Carswell et al. first described it. A cytokine displayed significant cytotoxic activity when the immune system was stimulated. This then would cause tumor necrosis. In 1984, when the gene was cloned, lymphotoxin (LT-alpha) was found. Combining these two findings led to the discovery of tumor necrosis factor-alpha (TNF-alpha) [22].

Inflammatory cytokine tumor necrosis factor-alpha (TNF-α) is a well-known commodity in pain management. TNF-α can modulate both inflammatory and neuropathic hyperalgesia through inflammatory cytokines [23]. The receptors for TNF-α are in both neurons and glial cells. The TNF-alpha action mechanism can be broken down into tumor necrosis factor receptor 1 (TNFR1) and tumor necrosis factor receptor 2 (TNFR2). TNFR1 is the receptor that initiates inflammatory responses and degenerative cascades.

On the other hand, TNFR2 is the response to anti-inflammatory and cytoprotective effects. TNF-alpha can also be divided into soluble TNF-alpha and transmembrane TNF-alpha. Soluble TNF-alpha binds to TRNF1 and provides inflammatory effects, while the transmembrane TNF-alpha binds to TRNF2 and provides anti-inflammatory effects. The soluble TNF-alpha to TRNF1 binding leads to NFxB and MAPK pathways, which I will discuss in greater depth later in the review [23,24].

The current usage of TNF-α implies that this mechanism mediates central mechanisms of neuropathic pain using glial systems. When nerve injury and inflammation are present, microglia can secrete these pro-inflammatory cytokines, including TNF-α, which, via the p38-MAPK pathway, can produce its own G-protein coupled receptor and TNF-α converting enzyme [4,23].

#### 3.1.2. Interleukin (IL-1β, IL-6)

Interleukin-1Beta is another potent pro-inflammatory cytokine important for injury defense and responses. There are 11 IL-1 family members, but I will outline the main ones involved in inflammatory pathways [25].

Interleukins can interact with the human body through autocrine, paracrine, and endocrine effects. Interleukin IL-1Beta and IL-6 are the ones we focus on in this review as they are directly involved in inflammatory conditions [26]. However, there are tons of different interleukins with various functions. Looking closer at IL-1, we see these consist of macrophages, large granular lymphocytes, B cells, and astrocytes. The main targets are T and B cells, crucial factors in inflammation manifestation. Therefore, understanding how to inhibit IL-1Beta, as mentioned in this review, is an integral part of testing the efficacy of the phytochemicals [27].

For IL-6, T and B lymphocytes are the main contributors, in addition to fibroblasts and macrophages [28]. The main targets for this interleukin are going to be lymphocytes and hepatocytes. Similarly to IL-1, IL-6 also plays a direct role in cases of inflammation. When determining and assessing the effect of phytochemicals on mitigating and reducing inflammation in patients or rodent models, inhibition of IL-1Beta and Il-6 is essential to optimal results [27].

### 3.2. Inflammatory Enzymes

#### 3.2.1. Cyclooxygenase (COX)

Cyclooxygenase is essential in the production of prostaglandins. Prostaglandins are heavily involved in hyperalgesia and its inflammatory effects. COX is a rate-limiting enzyme that plays a crucial part in creating prostaglandins. The COX enzyme is found in two different forms: COX-1 and COX-2. COX-2 is more relevant for this review as it is heavily involved in inflammation [29]. COX-2 is the inducible isozyme form expressed in inflammatory cells and tissues when inflammation is present. Thus, hyperalgesia is produced as a result. COX-2, in normal physiological conditions, will be in the kidney at low levels; however, it is highly inducible in the presence of inflammation. COX-2 converts arachidonic acid to PGG2 and then to PGH2, which is then converted to tissue-specific synthases [29,30].

Additionally, COX-2 selective inhibitors are potent against antihyperalgesic agents. It was shown in rat models that COX-2 specific inhibitor, coxibs, can reduce pain symptoms in rat models. COX-2 is also essential for central sensitization after peripheral inflammation, as seen in the rat models in the same studies [4,31].

#### 3.2.2. Nitric Oxide Synthase (NOS)

Nitric oxide is a colorless gas that plays a huge role in inflammation. Some of the earliest studies showed that nitric oxide was only pro-inflammatory; however, as more studies have emerged, NO’s anti-inflammatory capabilities have become more apparent. Nitric oxide can be synthesized using nitric oxide synthase. However, nitric oxide synthase also plays a significant role in nociceptive signaling. Using the activation of different isoforms of NOS, NOS plays a vital role in controlling hyperalgesia. It mediates neuronal excitotoxicity by activating the receptors downstream of MAPK signaling pathways. iNOS, the isozyme form, is expressed in glial cells. The iNOS form of NOS is the more critical form of inflammation. iNOS can provide inflammatory and anti-inflammatory effects. For inflammatory pain, iNOS can release some of the previously mentioned pro-inflammatory cytokines, TNF-alpha and IL-1Beta, and sensitize nociceptors, thus leading to inflammation. However, in lower levels, iNOS can regulate the pro-inflammatory cytokines and provide anti-inflammatory effects [30,32].

Pro-inflammatory cytokines such as TNF-alpha and IL-1Beta induce the expression of iNOS in microglia. This is carried out by activating the NF-kB pathway, which will be discussed later in this review [4,31,33].

Nitric oxide is critical in many physiological functions, including immunomodulatory and neuroinflammatory issues. Focusing on NOS signaling cascades will be a point of interest in the future, and more research surrounding phytochemical usage in this area should be conducted [34].

### 3.3. Inflammatory Pathways

#### 3.3.1. Nuclear Factor Kappa B (NF-kB)

The nuclear factor kappa B (NF-kB) pathway can regulate many genes involved with inflammatory and immune responses. The immune reactions this system can involve are infections, tissue injuries, or reactive oxidative species generation. The type of stimulation can also affect the NF-kB pathway. The canonical method of NFxB is the primary method for inflammation, which we will discuss in this review, as it uses TNF-alpha and IL-1Beta. The canonical path of NFxB is set into action by pro-inflammatory cytokines such as TNF-alpha and IL-1Beta. Thus, this leads to the expression of proinflammatory [35,36]. Many phytochemicals directly use this pathway, which regulates pro-inflammatory cytokines [37]. Interestingly, NFxB can contribute to leukocyte apoptosis, thus giving it anti-inflammatory qualities [35]. However, more research should be conducted to confirm this result. The activation of transcription factor NF-κB during hyperalgesia generally follows the canonical pathway [4,31].

#### 3.3.2. MAPK

MAPK pathways are generally associated with cell survival and death; however, there are also pain management applications for hyperalgesia. Similarly to NF-kB, the MAPK pathway also utilizes pro-inflammatory cytokines. The standard MAPK pathway consists of three kinases that will phosphorylate and activate each other in succession [38]. The outcomes are like the previous path: the MAPK pathway operates with TNF-alpha and interleukins IL-1Beta and IL-6. It can also regulate inflammatory mediators, and the inhibition of this pathway is tested concerning phytochemicals to see if optimal results are possible. MAPK heavily relies on signal transduction and downstream effects. The inhibitory effects of the phytochemicals are tested to determine whether they inhibit certain aspects of the pathway and thus affect the cascade to yield the desired result [37]. MAPK and NFxB pathway inhibition are the main goals of phytochemical application in chronic pain management, as the inflammatory mediators and nociceptive pain receptors work within these two systems.

## 4. Future Use and Overall Findings

Many inflammatory mediators currently being studied have been displaying promising results. It is also important to note that naturally occurring treatment options are more beneficial than synthetic ones [39]. Understanding the mechanisms and pathways the inflammatory mediators use is crucial to understanding how phytochemicals can impact pain management.

The research surrounding phytochemicals seems promising but requires large-scale clinical studies to understand their role in treating chronic pain better. However, the authors submit that phytochemicals need to be used on more diverse and larger populations to confirm preliminary findings. Phytochemical side effects and their safety profile with pharmaceutical combination therapy need to be studied to assess their role in chronic pain care more accurately [4].

## Figures and Tables

**Table 1:** This table differentiates the type of phytochemical present as well as the main components. The grouping of phytochemicals in the table can allow for contrast and comparisons between each other. The components associated with each phytochemical are those of primary significance and include various inflammatory mediators (proinflammatory cytokines, inflammatory enzymes, and inflammatory pathways).

Phytochemicals	Type	Effects
Curcumin	Polyphenol	Inhibits the COX-2, NF-kB, inducible NOS, and NO enzymes in specific macrophages and natural killer cells
Resveratrol	Polyphenol	Can inhibit NF-kB and TNF-alpha
Querecetin	Polyphenol	Inhibition of nociceptive effects of IL-1Beta and TNF-alpha
Naringenin	Polyphenol	Modulation of ROS and raises superoxide dismutase levels
EGCG	Polyphenol	Decreases IL-6, TNF-alpha, and ROS
Eugenol	Terpenoid	Reduced inflammation markers of COX-2, iNOS, TNF-alpha, and antioxidant enzymes
Capsaicin	Alkaloid	Desensitization of TRPV1 receptors
